# Spiliotis-Farfarelos Maneuver for the Management of Small Bowel Obstruction and Frozen Pelvis in Patients With Peritoneal Metastasis

**DOI:** 10.7759/cureus.32518

**Published:** 2022-12-14

**Authors:** John Spiliotis, Anastasia Prodromidou, Nikolaos Kopanakis, Christos Iavazzo, Christos Farfarelos

**Affiliations:** 1 Department of Surgical Oncology and HIPEC (Hyperthermic Intraperitoneal Chemotherapy), European Interbalkan Medical Center, Thessaloniki, GRC; 2 Department of Surgery, Metaxa Memorial Cancer Hospital, Piraeus, GRC; 3 Department of Obstetrics and Gynecology, Metaxa Memorial Cancer Hospital, Piraeus, GRC

**Keywords:** surgical maneuver, cytoreduction, advanced gynecological cancer, gastrointestinal cancer, peritoneal carcinomatosis

## Abstract

The management of the advanced peritoneal disease is demanding especially in cases of extensive bowel infiltration and the obstruction of the gastrointestinal tract in different sites. Patients with bowel obstruction due to peritoneal carcinomatosis have an overall survival that ranges from three to eight months to four to five weeks based on the operability or not of the disease, respectively. The decision to operate should carefully consider the balance between the probability of symptomatic relief and the risk of severe perioperative complications and survival after surgery. The extent of the disease and postoperative malnutrition could further complicate patients' postoperative course. We aim to present an operative maneuver of bowel preparation and fixation in cases of extensive infiltration of the small bowel by peritoneal carcinomatosis (PC) in order to eliminate the risk of postoperative fistula formation or anastomotic leakage.

## Introduction

Peritoneal carcinomatosis (PC) defined as the dissemination of malignant cells from various primary origins to the peritoneal cavity is considered a part of metastatic disease and thus requires respective management [1]. Palliative treatment is a choice for patients with metastatic peritoneal disease, while extensive debulking procedures when complete resection of the macroscopic lesions is feasible could also be considered [1].

Resection of peritoneal metastasis has been shown to improve survival in patients with peritoneal metastatic disease from gastrointestinal or gynecological cancer. The Peritoneal Cancer Index is a tool used at initial surgical diagnosis for the identification of the extent and resectability of the disease as well as the prediction of the overall disease prognosis [2]. The management of advanced peritoneal disease, especially in patients with a Peritoneal Cancer Index (PCI) greater than 15, is demanding due to the extensive infiltration of the bowel loops and the obstruction of the gastrointestinal tract in different sites. In particular, the proposed PCI cut-off for patients who could be potential candidates for cytoreductive procedures has been proven to be <25 and <20 for ovarian and colorectal cancers, respectively [3]. On the contrary, infiltration of the root of the small bowel mesentery and extensive small bowel disease that could leave short bowel syndrome after resection are potential contraindications for primary cytoreduction [3]. Furthermore, bowel obstruction complicates a significant proportion of patients with peritoneal carcinomatosis (PC), while as high as 50% of them presented with the recurrent disease even after complete cytoreduction [4]. Bowel obstruction can be due to either infiltration of the bowel and mesentery by the primary tumor or secondary due to the compression of the bowel from the primary lesion [4]. Obstruction can also be caused by the presence of cancer adhesions and carries a grim outlook. Patients with bowel obstruction due to PC have an overall survival that ranges from three to eight months to four to five weeks based on the operability or not of the disease, respectively [5]. In cases of PC with intestinal involvement, most of them include multiple metastatic sites mostly identified in the small bowel and are located in the pelvic part of the bowel and more specifically in the terminal ileum as well as the right colon. The surgical approach has been applied either for palliative purposes to reduce symptomatology or with curative intention in cases of malignancies where complete resection of the recurrent disease has been proved to improve survival [4]. Furthermore, the concomitant application of hyperthermic intraperitoneal chemotherapy (HIPEC) can offer a favorable therapeutic outcome in selected cases. Therefore, the decision to operate should carefully consider the balance between the probability of symptomatic relief and the risk of severe perioperative complications and survival after surgery. In particular, the extent of the disease and postoperative malnutrition could further complicate patients' postoperative course [5]. The majority of patients presented with malignant bowel obstruction may require extensive surgical procedures due to disease volume and previous surgeries that are associated with high morbidity with extensive dissections that can result in bowel openings or serosal laceration.

The purpose of the present study was to present an operative maneuver of bowel preparation and fixation in cases of extensive infiltration of the small bowel by PC. The technique was conceptualized by two surgeons aiming to eliminate the risk of postoperative fistula formation or anastomotic leakage.

## Technical report

**Figure 1:**
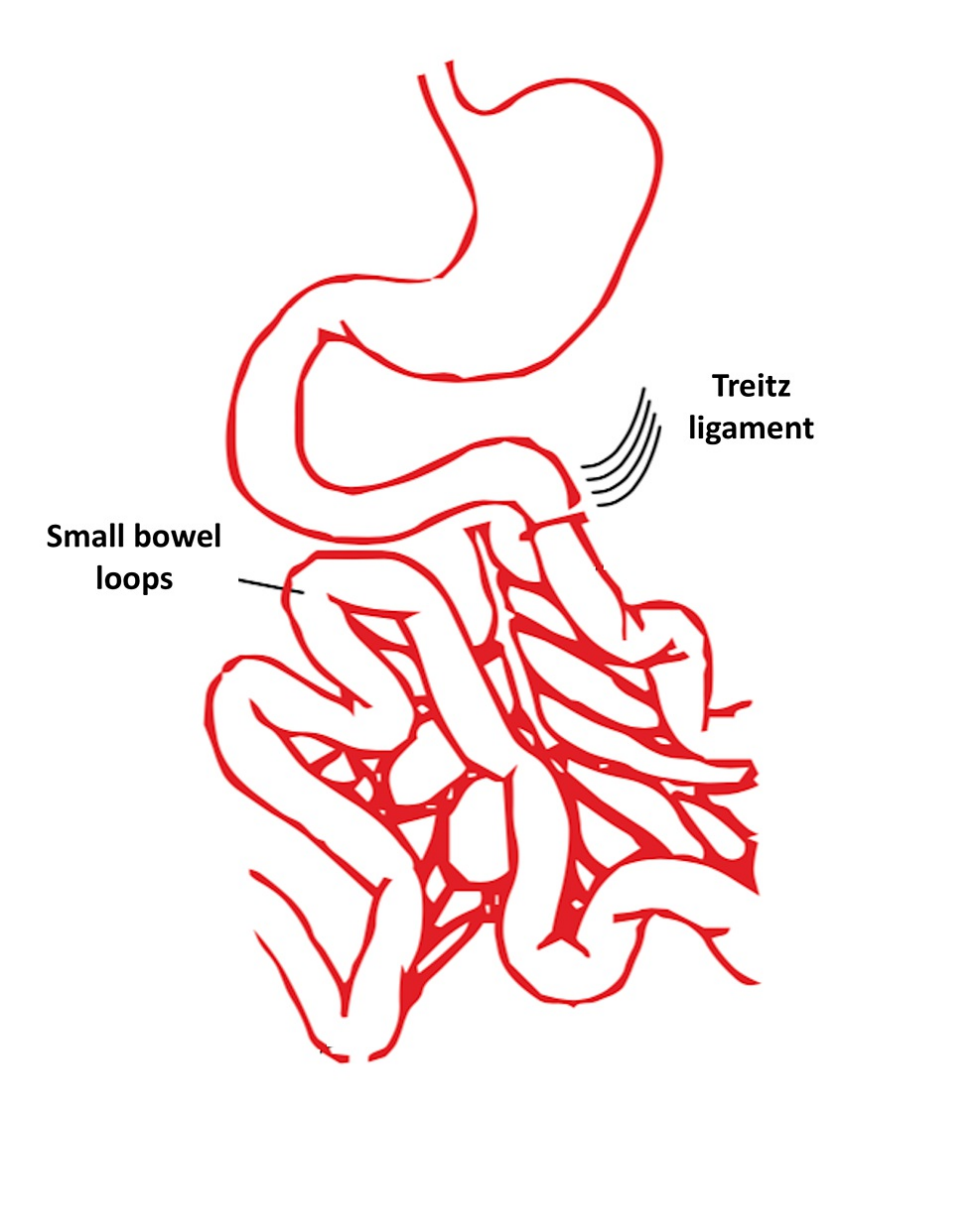
Recognition of the ligament of Treitz.

We present the “Spiliotis-Farfarelos” maneuver for the management of peritoneal metastasis that is complicated by small bowel obstruction. More specifically, the inclusion criteria were as follows: patients age ≤65 years, PCI ≤25, albumin levels ≥3.5g/dL, high-grade histological tumor types, and no liver metastasis. The maneuver is indicated in patients with frozen pelvis due to previous radiotherapy or pelvic tumors with peritoneal carcinomatosis with involvement of the small bowel and right colon. Regarding the surgical technique, the first step in the small bowel preparation is the recognition of the Treitz ligament (Figure 1).

**Figure 2:**
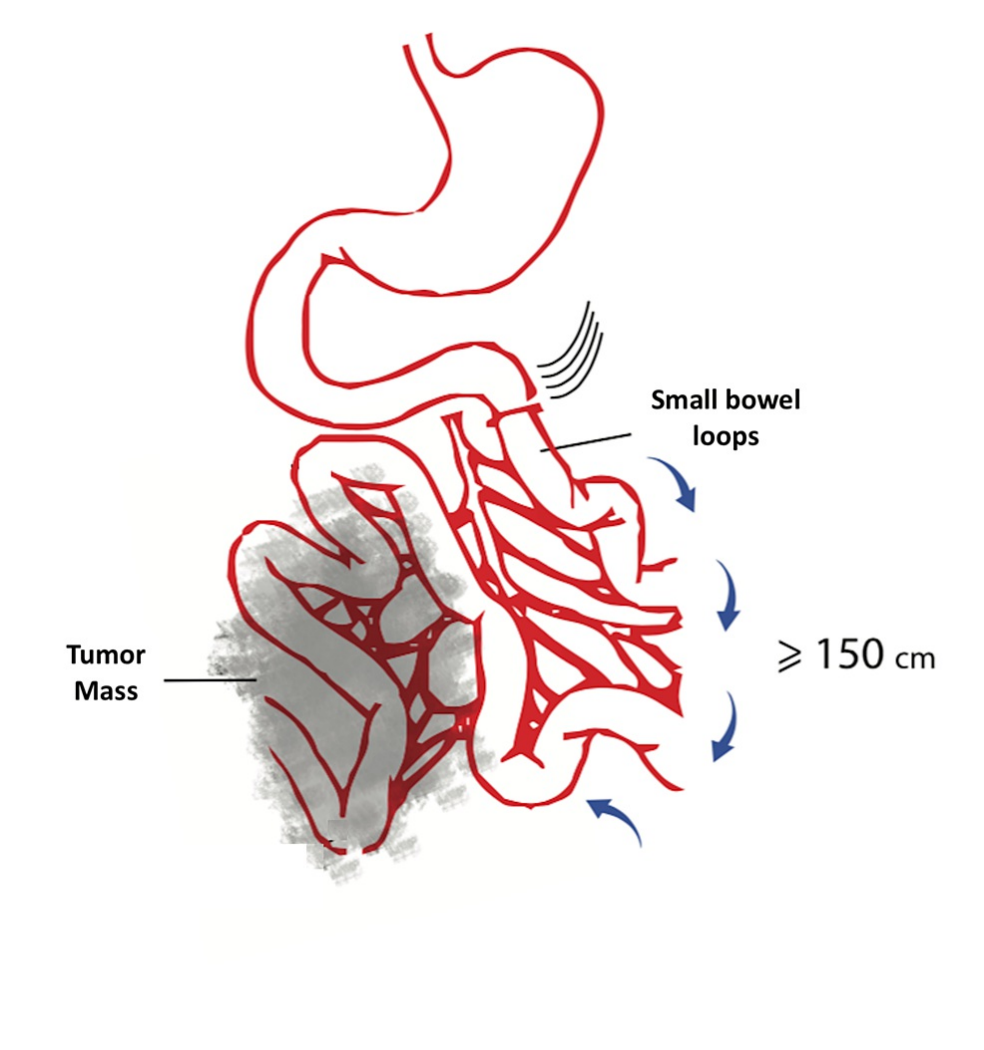
Identification of the small bowel loops that are located ≥150 cm from the Treitz ligament. Blue arrows indicate the inspection and preservation of the first loops of the small bowel (150 cm).

The small bowel loops are then followed till the measurement of more than 150 cm from the Treitz ligament (Figure 2).

**Figure 3:**
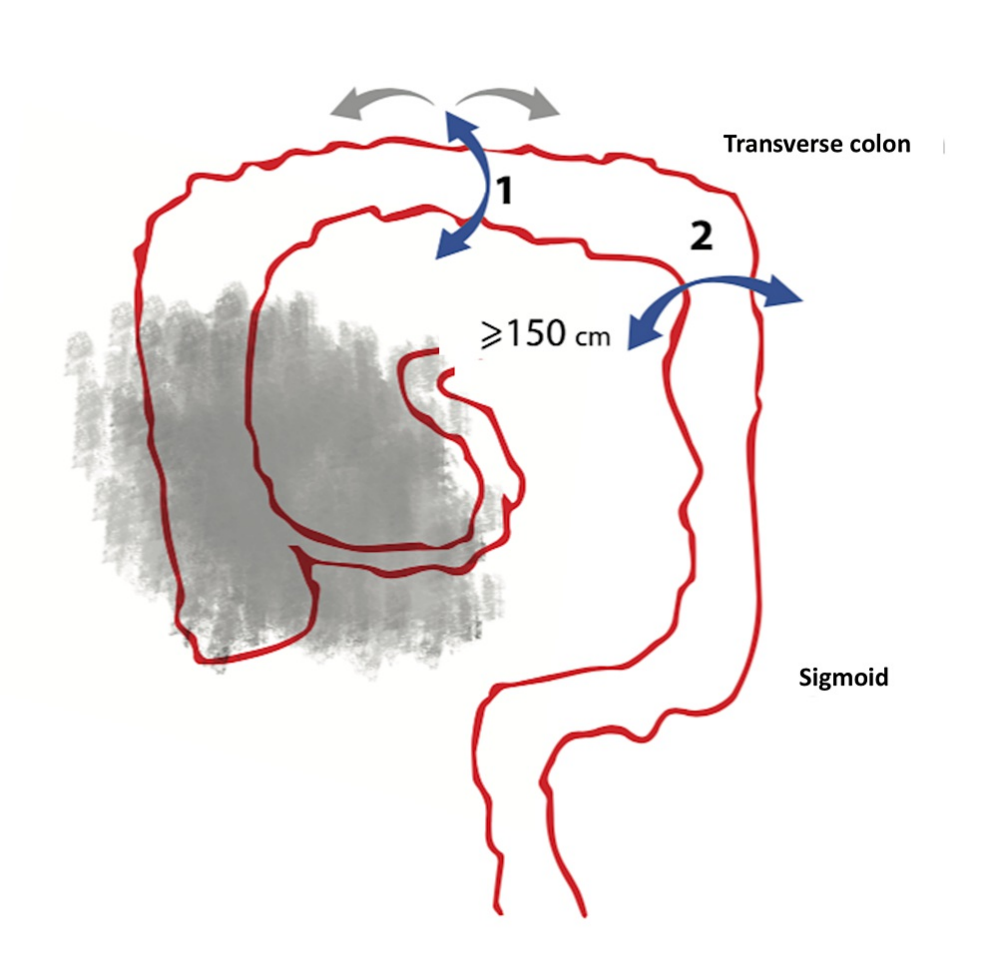
Preparation of the colon from the middle part of the transverse colon to the rectum. Blue arrow 1 indicates the mobilization of the transverse colon prior to transection. Blue arrow 2 indicates the potential mobilization of the splenic flexure in order to facilitate the jejunal-transverse anastomosis.

The second key step is to recognize the middle part on the left side of the transverse colon and to mobilize the transverse colon, rectosigmoid curve, and rectum, as shown in Figure 3.

**Figure 4:**
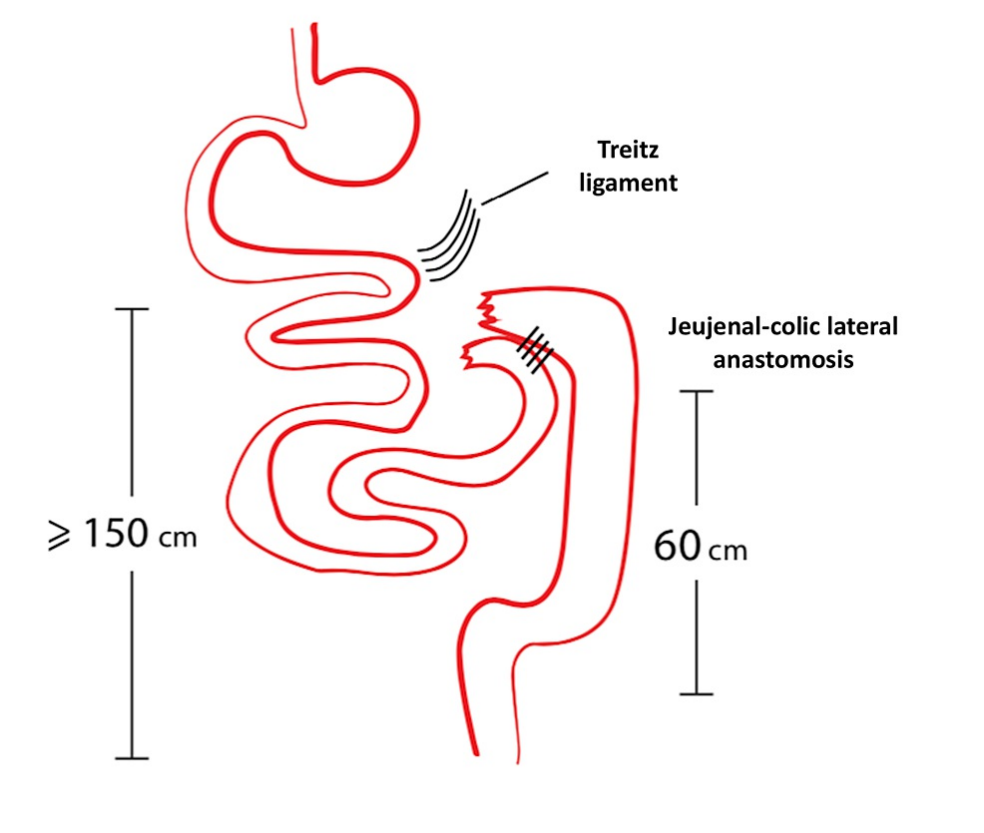
Removal of the affected small bowel along with the right and transverse colon and side-to-side jejunocolic anastomosis.

At the final step of the procedure, we remove the infiltrated part of the small bowel, the right colon, and the first middle part of the transverse colon. The procedure is completed with the jejunocolic side-to-side anastomosis with the use of a gastrointestinal (GIA) stapler that is inserted into each bowel end and aligned in the antimesenteric border (Figure 4). The preservation of the jejunum and left colon that are tumor free is of critical importance.

The maneuver was applied in 300 cases during an interval of 12 years (from 2008 to 2020) in patients with small bowel obstruction due to peritoneal metastatic disease. Table 1 depicts the main characteristics of the included patients and the main perioperative outcomes.

**Table 1:** Baseline patient characteristics and main outcomes. ^a^Median (range). PCI: Peritoneal Cancer Index.

Baseline patient characteristics and main outcomes	
Age (years)	56.8 (29-63)^a^
Type of malignancy N (%)	
Ovarian cancer	180 (60)
Colorectal cancer	60 (20)
Peritoneal mesothelioma	25 (8.3)
Gastric cancer	20 (6.7)
Other	15 (5)
PCI	16 (6-28)^a^
Completeness of cytoreduction	
R0 resection N (%)	201 (67)
R1 resection N (%)	99 (33)
Operative time (min)	290 (160-410)^a^
Length of stay (days)	10 (7-23)^a^
30-day morbidity N (%)	63 (21)
30-day mortality N (%)	8 (2.7)
Overall survival (months)	26.4

The most common primary tumor was ovarian cancer (OC) in 180 patients. R0 resection was achieved in 67% of patients. The median operative time was 290 min. Postoperative care was the standard that we follow for patients who have extensive cytoreductive procedures including bowel resections. The median hospital stay was 10 days. Thirty-day morbidity and mortality were 21% and 2.7%, respectively. The most common postoperative complications were enterocutaneous fistula in 54 patients (18%) and pulmonary embolism in 18 patients (6%). On a long-term basis, the cases of short bowel syndrome were coped with special oral intake and Home Parenteral Nutrition (HPN) two times per week. The application of the technique offered a median survival prolongation of 10 months when compared with patients who received palliative supportive care (26.4 months vs 15.2 months) [6].

## Discussion

We herein describe a surgical maneuver for the management of bowel obstruction caused by peritoneal dissemination of advanced peritoneal malignancies. Among the steps of the procedure, the recognition of the Treitz ligament, the following loops of the small and large bowel until the level of the transverse colon, and the subsequent preparation of the left middle of the transverse colon and left colon until the level of rectum play a key role in the resection of the disease.

The main goal during cytoreductive surgery is to achieve complete cytoreduction and to eliminate all the visible diseases which have been related to improve overall survival, especially in patients with ovarian and colorectal cancers. Additionally, decreasing the number of intestinal anastomoses as well as the risk of fistulas or leaks is also considered of critical importance. The Spiliotis-Farfarelos maneuver represents an optimal surgical approach related to significant functional and anatomical outcomes. The maneuver could decrease the risk of gastrointestinal fistulas. More specifically, we tried to eliminate the serosal traumatic surfaces by avoiding the unnecessary "lysis" of adhesions in the blocked loops of the small intestine. It also provides the opportunity to decrease the number of gastrointestinal anastomoses which is considered the main reason for morbidity after aggressive cytoreductive procedures. Therefore, it can also contribute to the respective reduction of intensive care unit (ICU) stay and overall hospitalization. Short bowel syndrome is considered a critical and sometimes inevitable complication of such extensive bowel resections and can lead to significant malabsorption of macronutrients and specific micronutrients and malnutrition [7]. However, with the proposed maneuver, if we resolve 150 cm of the small intestine, the percentage of malnutrition is less than 15% and the malabsorption of vitamins can be resolved with oral nutritional supplements and home parenteral nutrition twice per month [7,8]. The main early complication is diarrhea within the first four to six weeks which can be managed medically with medication and special diet formulas [8,9]. We strongly believe that the technique is unique based on the fact that it enables the preparation of the bowel loops and the easiest en bloc resection of the compromised loops of the small bowel and right colon without even touching the affected area. This could make our final goal that is R0 resection more reproducible. Additionally, the technique refers to the resectable lesions with the aim to achieve no residual disease and completeness of cytoreduction. Therefore, bypass that refers to palliative surgical procedures is not an option. Our technique is not indicated for palliative purposes and that is why we do not propose the bypass that leaves residual disease.

The detection of malignant bowel obstruction is based on the findings of clinical signs and symptoms and imaging evidence. Bowel obstruction due to peritoneal dissemination has been reported as a complication that occurs in 10%-30% of all colorectal cancers and 20%-50% of all ovarian cancer cases or other gynecological tumors [10,11]. In cases of peritoneal carcinomatosis, the majority of lesions are detected in the pelvis, followed by the central region of the bowel and the right upper and lower quadrants [11,12]. Because of the pathophysiological mechanisms, the diagnosis and treatment during the exploratory laparotomy may be challenging depending on the patient’s performance status and response to previous treatments.

Malignant bowel obstruction is associated with poor prognosis also indicating disease progression [5]. The management of those patients seems extremely challenging especially due to the emergent nature of the procedure that is associated with increased morbidity and mortality compared to elective operations [13]. The decision to proceed with the operation is difficult and should be based on the patient’s performance status and the symptoms caused by the obstruction [5,13]. Furthermore, an estimation of the patient’s life expectancy is also of paramount significance [5]. In most of the cases, the palliation of the symptoms is the main target of surgical intervention, while complete excision of the disease is complex and has been associated with increased morbidity and mortality.

Before reaching firm results, there are some limitations that need to be addressed. The appropriate pool of patients could not be achieved given the significant heterogeneity of the extent of peritoneal disease among patients with peritoneal carcinomatosis. More specifically, the described maneuver is only a part of cytoreductive procedures due to PC for the lesions that affect the small bowel. Independent of the aforementioned maneuver concerning small bowel resection, additional lesions in the remaining peritoneal cavity including those in the upper abdomen and the remaining infiltrated pelvic structures may need to be removed to achieve complete cytoreduction.

## Conclusions

Bowel obstruction due to peritoneal carcinomatosis represents a severe and challenging to handle scenario. The decision to proceed with resection should take into account the patient's current clinical condition, the life expectancy, and the potential increased morbidity and mortality associated with such extensive procedures. The Spiliotis-Farfarelos maneuver proposes that the key points in the resection of the disease are the recognition of the Treitz ligament, the following loops until the level of the transverse colon, and the subsequent preparation of the transverse and left colon until the level of the rectum. Based on our experience with the performance of this maneuver, patients benefit from significant functional and anatomical outcomes.
